# Assessment of cognitive function in elderly patients with heart failure

**DOI:** 10.1590/1806-9282.20240429

**Published:** 2024-08-16

**Authors:** Leandro Marques da Silva, Carla Priscilla Belchior Marques Sampaio, Nair Eloá dos Santos Guimarães, Luiza Pinto Moreno, Gedean Souza Pontes, Emmanuela de Jesus Furtado Ferreira, José Albuquerque de Figueiredo

**Affiliations:** 1 Universidade Federal do Maranhão, Doctoral Program in Health Sciences – São Luís (MA), Brazil.; 2 Universidade Federal do Maranhão, Master's Program in Adult Health – São Luís (MA), Brazil.; 3 Pitágoras College, Nursing Course – São Luís (MA), Brazil.; 4 Edufor College, Physiotherapy Course – São Luís (MA), Brazil.; 5 Universidade de São Paulo – São Luís (MA), Brazil.

**Keywords:** Cognition, Neuropsychological tests, Heart failure

## Abstract

**OBJECTIVE:**

To compare the Mini-Mental State Examination (MMSE) and Montreal Cognitive Assessment (MoCA) tests for the identification of cognitive deficit (CD) in elderly patients with heart failure (HF).

**METHODS:**

This was a cross-sectional study with an observational design involving 43 elderly patients with HF of both sexes, treated by the Unified Health System, who were able to understand and follow the study instructions. A sociodemographic and clinical questionnaire and the MMSE and MoCA neurocognitive tests were applied.

**RESULTS:**

The mean age of the patients was 67 years; 67.44% were male; 53.49% were white; 58.14% had 1–4 years of schooling; 58.14% had an income of half to one minimum wage; 55.81% were married; 53.49% had a family history of HF; 90.7% denied smoking; 83.72% denied alcohol intake; 65.12% did not practice physical activity; 83.72% were hypertensive; 30.23% were diabetic; 57.89% had LVEF ≥ 50%; 39.53% have NYHA II; and 88.37% did not have a pacemaker. In the identification of CD, the MMSE test detected it in 25.58% of the patients, while the MoCA test identified it in 23.26% (p=0.043).

**CONCLUSION:**

It was concluded that the MMSE test performed better than the MoCA test in the identification of CD in elderly patients with HF.

## INTRODUCTION

Heart failure (HF) is a complex clinical syndrome resulting from structural or functional problems affecting ventricular filling or blood ejection. This condition compromises the heart's ability to supply sufficient oxygen to tissues to meet their metabolic needs^1,2^.

Although likely underestimated, the prevalence of HF is estimated to be between 1 and 2% of the general adult population. It affects 6.5 million Brazilians and 5.7 million Americans. According to estimates, the prevalence of HF will increase by 46% between 2012 and 2030, resulting in over 8 million people with HF in Brazil, mainly due to population aging^1^.

Studies have demonstrated common triggers between cardiovascular diseases and dementia, such as inflammation, oxidative stress, oxygen deprivation, and adrenergic signaling^3,4^. Maintaining normal brain function requires a constant supply of metabolites, which depends on proper heart function. As a systemic disease, HF can damage other organs, including the brain^5^.

Neuropsychological tests are frequently used to detect brain dysfunction, such as cognitive deficit (CD), and evaluate performance in different cognitive areas, including learning and memory, language, visuospatial abilities, executive function, and psychomotor function. CD is defined as the decline or loss of at least one of these five domains^6^.

Currently, there are no well-defined guidelines for cognitive screening, and standardized cognitive screening tests can determine the prevalence of CD in older adults with HF. Early detection of cognitive changes allows for rapid intervention through multidisciplinary follow-up, preventing the progression of functional impairment in the HF population.

Therefore, this study aims to compare the Mini-Mental State Examination (MMSE) and Montreal Cognitive Assessment (MoCA) tests for identifying CD in older adults with HF.

## METHODS

This is a cross-sectional study with an observational design, conducted with 43 elderly patients with HF, seen at the Cardiology Outpatient Clinic of the University Hospital of the Federal University of Maranhão (HUUFMA), in the city of São Luís, MA. The research was carried out between February and December 2022 and was previously approved by the HUUFMA Ethics and Research Committee (Opinion No.: 3.902.939/CAAE: 24168819.2.0000.5086).

Patients with HF of both sexes who were 60 years of age or older, NYHA functional classes I to IV, able to comprehend and adhere to the study's instructions, and who consented to participate by signing the Informed Consent Form (TCLE) were included. The European Society of Cardiology's (2021) guidelines were taken into consideration for diagnosing heart failure (HF).

Patients with chronic atrial fibrillation; acute decompensation of HF, with a clear history of central nervous system injury, such as trauma, tumor, infection, carbon monoxide poisoning, and demyelinating disorders; alcohol abuse (as measured by the CAGE questionnaire); use of drugs or psychoactive substances (as measured by the ASSIST instrument) that can demonstrably cause changes in the nervous system and cognition; and disorders related to hearing, reading, language expression, or writing were excluded.

The following sociodemographic data were collected: age, sex, race, income, education, marital status, and clinical data such as family history of HF, ejection fraction (EF), functional classification (NYHA), and the presence of comorbidities such as hypertension and diabetes, history of smoking, alcohol consumption, and physical activity practice.

A neuropsychological assessment was also performed by a psychologist to identify CD. The MMSE test was initially applied; it has strong reliability and internal consistency, and its use is validated and recommended in Brazil, which enables a rapid assessment of cognitive functions by examining both verbal and non-verbal forms of responses.

The MMSE score ranges from 0 to 30, with higher scores indicating better cognitive performance. The test results in this study were adjusted according to the individual's education level, as described by Brucki et al^7^, applying the following cut-off criteria: 20 points for illiterate patients; 25 points for those with 1–4 years of study; 26.5 points for 5–8 years; 28 points for 9–11 years; and 29 points for more than 11 years.

In addition, the MoCA test, validated for the Portuguese language, was used. A point is added to the maximum total possible score of 30 points if the individual has less than 12 years of education. In this study, the following cut-off scores were considered for the detection of CD: illiterate, score ≤ 11; 1–4 years of schooling, score ≤ 17; 5–8 years of schooling, score ≤ 19; 9–11 years of schooling, score ≤ 19; and ≥ 12 years of schooling, score ≤ 21^8^.

Microsoft Office Excel (version 365) was used to tabulate the data, and R Studio (R Core Team, 2021^®^) was used for statistical analysis. The normality of the continuous variables was initially examined using the Shapiro-Wilk test. The description of the continuous data was given by medians and interquartile ranges (IIQ), while the categorical variables were described in simple frequencies (n) and percentages (%). The relationship between categorical variables was established using Fisher's exact test. Subsequently, the Spearman Correlation test was performed to evaluate the existence of proportionality between the continuous variables that were being studied. Statistical significance was set at p<0.05.

## RESULTS

The sociodemographic and clinical data of 43 elderly patients with HF were analyzed. The mean age was 67 years, 67.44% were male, the white race was predominant (53.49%); 58.14% had 1–4 years of education; 58.14% had an income of half to one minimum wage; 55.81% were married; 53.49% had a family history of HF; 90.7% were non-smokers; 83.72% did not drink alcoholic beverages; 65.12% did not practice physical activity; 83.72% were hypertensive; and 30.23% were diabetic. Regarding clinical data, 57.89% had EF ≥ 50%; 39.53% NYHA II, and 88.37% did not have a pacemaker (Table 1).

**Table 1:** Sociodemographic characteristics and clinical data of elderly patients with heart failure in São Luís, MA, Brazil, 2022.

Variable		n=431
Age		67.00 (63.50, 73.50)
Gender		
	Female	14 (32.56%)
	Male	29 (67.44%)
Ethnicity/color		
	White	23 (53.49%)
	Black	8 (18.60%)
	Brown	12 (27.91%)
Educational level		
	Incomplete elementary school	25 (58.14%)
	Elementary school graduate	5 (11.63%)
	High school graduate	10 (23.26%)
	Incomplete bachelor's degree	1 (2.33%)
	Bachelor's degree	2 (4.65%)
Income		
	Less than ½ minimum wage	3 (6.98%)
	½ to 1 minimum wage	25 (58.14%)
	1 to 2 minimum wages	12 (27.91%)
	2 to 5 minimum wages	3 (6.98%)
Marital status		
	Single	8 (18.60%)
	Divorced	5 (11.63%)
	Married	24 (55.81%)
	Widowed	6 (13.95%)
HF family history		
	Yes	23 (53.49%)
	No	20 (46.51%)
Smoker		
	Yes	4 (9.30%)
	No	39 (90.70%)
Drinks alcohol		
	Yes	7 (16.28%)
	No	36 (83.72%)
Engages in physical activity		
	Yes	15 (34.88%)
	No	28 (65.12%)
Hypertension		
	Yes	36 (83.72%)
	No	7 (16.28%)
Diabetes		
	Yes	13 (30.23%)
	No	30 (69.77%)
Pacemaker		
	Yes	5 (11.63%)
	No	38 (88.37%)
LVEF (%)		
	<40%	11 (28.95%)
	40 to 49%	5 (13.16%)
	≥50%	22 (57.89%)
NYHA		
	I	10 (23.26%)
	II	17 (39.53%)
	III	13 (30.23%)
	IV	3 (6.98%)

Median (IQR); n (%); HF, heart failure; NYHA, New York Heart Association; LVEF, left ventricular ejection fraction.

In a comparative analysis of the applied tests, considering the cut-off points for the screening of CD (based on educational level), it was observed that MoCA identified CD in 23.26% of the sample and MMSE identified CD in 25.58% of the sample.

Regarding the distinction between patients with and without CD, there was a statistically significant difference between the two tests (Table 2).

**Table 2:** Comparison between Mini-Mental State Examination and Montreal Cognitive Assessment tests in the identification of cognitive deficit in elderly patients with heart failure in São Luís, MA, Brazil, 2022.

	MMSE (n=43)	MoCA (n=43)	p
Cognitive deficit	11 (25.58%)	10 (23.26%)	0.043
No cognitive deficit	32 (74.42%)	33 (76.74%)	

MMSE: Mini-Mental State Examination; MoCA: Montreal Cognitive Assessment.

There was a strong correlation between the neurocognitive test scores in identifying CD in the analyzed patients (p<0.001), as described in Table 3.

**Table 3:** Correlation between the scores of the applied tests in elderly patients with heart failure in São Luís, MA, Brazil, 2022.

	Coefficient	p
Score MMSE vs. score MoCA	0.786	<0.001

MMSE: Mini-Mental State Examination; MoCA: Montreal Cognitive Assessment.

## DISCUSSION

In line with the current study's findings, an analysis of 545 medical records of patients with heart failure (HF) receiving treatment from the Unified Health System (SUS) showed that 55.6% were male, 76.7% had hypertension, and 37.2% had diabetes^9^. According to the National Health Survey (2019), low education and income between half and one minimum wage are consistent with the sociodemographic profile of SUS care^10^.

It was observed that 57.89% of the sample had EF ≥ 50%, classifying the patients as heart failure with preserved ejection fraction (HFpEF). Additionally, mild symptoms (NYHA II) were assigned to 17 patients (39.53%).

The diagnosis of systemic arterial hypertension (SAH) was found in 83.72% of the sample, confirming the significance of this condition for the development of HF, in addition to family history also being relevant for this. SAH is one of the primary causes of HF in Brazil^11^. Hypertension acts in the pathophysiology of cognitive impairment through neurodegeneration^12^. Thus, there is an association between SAH, HF, and cognitive decline.

Table 2 indicates that there was a statistically significant difference between the tests that were used, which has been observed in other studies including patients with SAH, cerebrovascular disease^13^, and HF^14^. In the comparison between the tests applied in patients with HF, the MMSE obtained a higher prevalence of cognitive decline (25.58%) compared to the MoCA (23.26%), with a statistically significant difference.

In a study that evaluated both tests in 106 patients diagnosed with HF and with a mean age of 68 years, it was observed that the MMSE detected cognitive decline in 68% while the MoCA test in 65% of the sample^15^. Although the prevalence of cognitive deterioration identified in the present study is lower than that predicted in the literature, these results support the findings of the present study, which similarly focused primarily on patients with HF.

A systematic review^16^ showed that in the vast majority of articles analyzed, the MoCA was superior to the MMSE in detecting individuals with mild cognitive impairment (MCI), but both were similar in detecting Alzheimer's disease. In a different study, 93 hospitalized patients with HF and a mean age of 70 years were studied. It was observed that the MoCA identified MCI in 41% more cases than the MMSE, indicating that the changes in the visuospatial dimension of the MoCA were clinically more significant than those found in a similar task in the MMSE^14^.

In a cross-sectional analysis of the Chinese population>55 years of age, the MoCA performed better than the MMSE, particularly in the identification of MCI, with 36.2% versus 28.6% of the sample^17^. However, in this sample, only 31.8 and 1.9% reported a history of hypertension and acute myocardial infarction, respectively, which are among the leading causes of HF in the world^11^. In the present study, 83.72% of the individuals had a history of hypertension, which may perhaps justify the discordant findings between the two studies.

Another factor for the MMSE's better performance in detecting cognitive decline may be linked to the low educational level of the sample in the present study (50.14%). In turn, the MoCA has a greater sensitivity in identifying cognitive decline in patients with higher educational levels^18^, thus justifying the results obtained.

This study had some limitations, such as the lack of sample size calculation, with the sample being obtained by convenience according to the cases seen at the outpatient clinic. Another factor is the predominance of low educational level among the patients evaluated, which could have affected the diagnosis of MCI because education is a variable that significantly affects both tests.

## CONCLUSION

The MMSE test performed better in detecting cognitive decline (CD) in elderly patients with heart failure (HF) compared to the MoCA test, possibly due to the low educational level of the sample analyzed. The application of neurocognitive screening tests is essential for the early identification of CD in patients with HF, aiming to provide appropriate treatment for patients.
